# Geographical distribution of two acoustic fin whale (*Balaenoptera physalus*) populations across the Weddell Sea

**DOI:** 10.1098/rsos.241866

**Published:** 2025-03-05

**Authors:** Svenja Wöhle, Karolin Thomisch, Elke Burkhardt, Ilse Van Opzeeland, Elena Schall

**Affiliations:** ^1^ Alfred Wegener Institute for Polar and Marine Research, Klußmannstraße 3d, 27570 Bremerhaven, Germany; ^2^ Helmholtz Institute for Functional Marine Biodiversity (HIFMB), Carl von Ossietzky University Oldenburg, Ammerländer Heerstraße 231, 26129 Oldenburg, Germany

**Keywords:** passive acoustic monitoring, acoustic populations, fin whales, Southern Ocean, conservation

## Abstract

Understanding and identifying population-specific acoustic features is crucial to passive acoustic monitoring-based remote sensing of population distributions. Fin whales are known to produce 20-Hz pulses, often accompanied by a simultaneous higher frequency (HF) component. The centre frequency of this component has been found to differ regionally, presumably representing a population-specific acoustic characteristic. Within the Southern Ocean, five distinct HF components have been identified so far, two of which are present in the Atlantic Sector of the Southern Ocean (ASSO) with peak frequencies around 86 and 99 Hz. This study investigates the extent to which these HF components indicate distinct acoustic fin whale populations and their spatial distribution across the ASSO. By automatically analysing passive acoustic data from 2013, across 10 recording positions, our data show that while the 99-Hz component was detected at seven recording positions throughout the ASSO, the 86-Hz HF component is only present in its western area, centred around the Western Antarctic Peninsula. Additional 2019 data from the Western Antarctic Peninsula confirmed the consistent presence of the 86-Hz component, suggesting that these components are robust indicators of distinct acoustic populations. Knowledge on population-specific key habitats is key to strategic and effective conservation efforts.

## 1. Introduction

Acoustic signals play a crucial role in the ecology of many animal species, serving functions ranging from echolocation for orientation to vocalizations for communication such as mating display (e.g. [1–4]). Like human language, animal communication often exhibits within-species vocal variations or ‘dialects’, offering valuable insights into the species’ ecology.

In the absence of genetic and morphological data, acoustic signals can in some cases indicate acoustic populations and support management decisions since understanding a species’ population structure and their spatial distribution is essential for developing targeted and effective conservation management strategies [5–9]. This is particularly relevant in logistically challenging environments where traditional monitoring methods are impractical. Here passive acoustic monitoring (PAM) methods are invaluable for long-term studies on the distribution of soniferous marine mammals, their main habitats and related behaviour [10–12].

For instance, regional differences in humpback whale (*Megaptera novaeangliae*) song were shown to reflect population identity and structure, as well as cultural exchange among populations (e.g. [13,14]). Similarly, the stereotyped song of several other cetacean species such as blue whales (*Balaenoptera musculus*), pygmy blue whales (*Balaenoptera musculus brevicauda*) and fin whales (*Balaenoptera physalus*) can be used to identify acoustic populations and their distribution [15–21].

This study focuses on identifying a reliable and easily recognizable acoustic cue to differentiate acoustic populations of Southern Hemisphere fin whales (SHFW) to contribute to knowledge vital to the implementation of targeted and successful conservation strategies. After decades of severe exploitation during the commercial whaling era, effective management measures are key to restore the SHFW stocks. However, the rarity of observations which is due to the difficult logistics of studying the species has led to data deficiency on the species’ habitat use, ecology, population structures and recovery rates (IWC-SORP, https://www.marinemammals.gov.au/sorp/southern-hemisphere-fin-whales/). Consequently, by lack of further data, the SHFW are currently managed as one circumpolar stock (IWC-SORP, https://www.marinemammals.gov.au/sorp/southern-hemisphere-fin-whales/).

Globally, fin whales are known to produce stereotyped 20-Hz pulses, characterized as short (approx. 1 s), loud impulse sounds (160–186 dB re 1 μPa at 1 m) centred around 20 Hz, with a frequency range typically sweeping from approximately 28 Hz down to approximately 15 Hz. These pulses occur both as single vocalizations and as song, produced exclusively by males. In contrast to the complex and hierarchical structure of humpback whale songs, fin whale song is less complex, consisting of repetitive and structured pulse sequences [22–24]. Mainly, fin whale acoustic populations are differentiated based on song characteristics such as the inter-note intervals (INIs; e.g. [20,25]) or the occurrence of ‘20-Hz pulse doublet calls’ [26], but also the high-frequency (HF) component accompanying the 20-Hz pulse has been suggested to be a valuable characteristic helping to identify acoustic populations [20,27,28]. In the Northern Hemisphere, one HF component was observed ranging between 125 and 130 Hz, not necessarily occurring simultaneous to the 20-Hz pulse or seeming to be reflective of geographical differences [12,20]. In contrast, in the Southern Ocean (SO) five presumably region-specific HF component varieties have been identified so far. These components range between 60 and 100 Hz and seem to consistently occur simultaneously with the 20-Hz pulses [10,11,18,26,27,29–32]. Two HF component varieties have been found present in the Atlantic Sector of the Southern Ocean (ASSO) with peak frequencies around 86 and 99 Hz [11,31,33]. The 99-Hz HF component was also detected throughout the Indo-Pacific Ocean towards the western coast of Australia, whereas different doublet HF components with peak frequencies at 82 and 94 Hz were found off the Australian east coast. Two further geographically separate doublet HF components were found in waters north and south of New Zealand with peak frequencies at 77 and 88 Hz and 67 and 73 Hz, respectively [26,27,34,35]. These different peak frequencies and the geographical distinction imply that the HF component varieties may represent different acoustic populations of SHFW [27,31,34]. Simon *et al*. [28] suggested that, in the SO, unlike in the Northern Hemisphere, the HF component possibly is a more reliable indicator than INI information to assess population identity, particularly in areas where songs and single vocalizations of multiple simultaneously vocalizing individuals spectrally and temporally overlap.

Given these findings, this study aims to further explore the regional specificity of the HF component and the potential to identify SHFW acoustic populations within the ASSO. We will improve the knowledge on SHFW (i) by investigating the acoustic presence and distribution of the HF components and the respective acoustic fin whale populations in the Weddell Sea by automatically analysing passive acoustic data from 2013; (ii) by comparing HF components from 2013 and 2019 data to assess their spectral stability; and (iii) by providing an overview on the distribution of additionally described HF components in the SO.

## 2. Material and methods

### 2.1. Passive acoustic data

**Figure 1.**
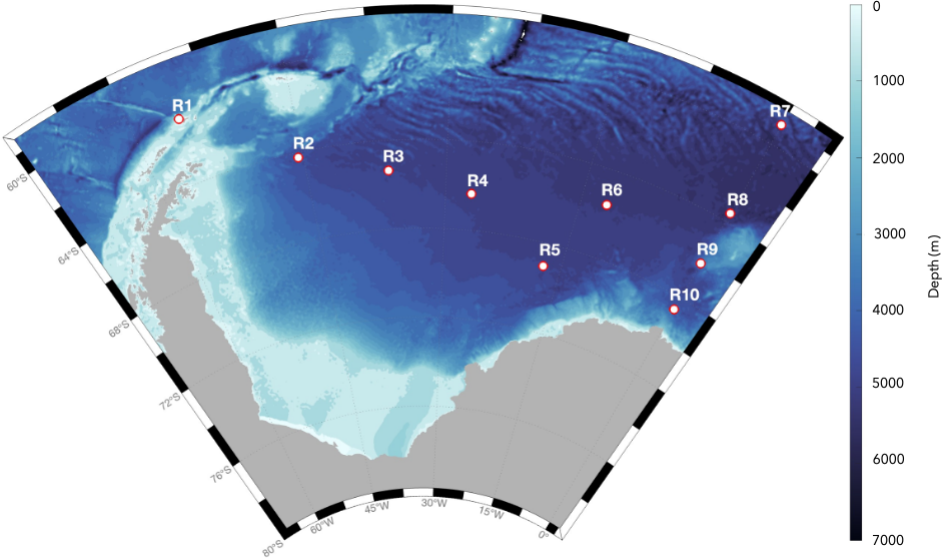
Bathymetric map of the Atlantic Sector of the Southern Ocean indicating the geographical locations of the 10 acoustic recorders (R1–R10) used in this study. Map was generated with M-MAP [36] in MATLAB [37].

Fin whale acoustic presence was investigated using passive acoustic data from 10 recording positions throughout the ASSO (figure 1). Data selection involved visual examination of long-term spectrograms not only to assess data quality but also to ensure the best possible spatial coverage, using all available Alfred Wegener Institute recorders within the ASSO, accessible through the Open Portal to Underwater Soundscapes (https://opus.aq/).

Acoustic data were obtained using SonoVault autonomous recorders (Develogic GmbH, Hamburg, Germany, Reson TC4037-3 hydrophone, with a linear frequency range of 1 Hz–50 kHz), which continuously recorded at a sampling frequency of 5333 Hz in 2013 (see table 1 for detailed deployment information). Preparation and standardization of passive acoustic data were implemented according to the standard operating procedures of the Ocean Acoustics Group at the Alfred Wegener Institute in Bremerhaven, Germany [48].

**Table 1.** Deployment information on passive acoustic recordings included in this study. Depth refers to recorder deployment depth. All recorders were operating with a sampling frequency of 5333 Hz, resulting in a bandwidth of 2666.5 Hz.

recorder	deployment ID	latitude	longitude	depth (m)	recording period	data citation
R1	AWI251−01_SV1008	61 0.88° S	55 58.53° W	212	15 Jan 2013–9 Nov 2013	https://doi.org/10.1594/PANGAEA.97313891 [38]
R2	AWI217−05_SV1020	64 22.94° S	045 52.12° W	960	23 Feb 2013–19 Jun 2013	https://doi.org/10.1594/PANGAEA.973149 [39]
R3	AWI208−07_SV1030	65 37.23° S	036 25.32° W	956	2 Jan 2013–20 Oct 2013	[40] https://doi.org/10.1594/PANGAEA.968560
R4	AWI209−07_SV1028	66 36.45° S	027 7.26° W	1085	31 Dec 2012–22 Oct 2013	[41] https://doi.org/10.1594/PANGAEA.973151
R5	AWI245−03_SV1012	69 3.480° S	017 23.32° W	1065	28 Dec 2012–11 Nov 2013	[42] https://doi.org/10.1594/PANGAEA.973236
R6	AWI248−01_SV1013	65 58.09° S	012 15.12° W	1081	18 Jan 2013–14 Nov 2013	[43] https://doi.org/10.1594/PANGAEA.973408
R7	AWI227−12_SV1025	59 2.82° S	000 5.78° E	1020	11 Dec 2012–13 Jul 2013	[44] https://doi.org/10.1594/PANGAEA.966612
R8	AWI229−10_SV1010	63 59.85° S	000 1.84° E	998	14 Dec 2012–2 Aug 2013	[45] https://doi.pangaea.de/10.1594/PANGAEA.973171
R9	AWI230−08_SV1009	66 2.01° S	000 3.12° E	949	7 Jan 2013–27 Sep 2013	[46] https://doi.pangaea.de/10.1594/PANGAEA.973185
R10	AWI232−11_SV1011	68 59.94° S	000 4.38° E	958	17 Dec 2012–13 Nov 2013	[47] https://doi.org/10.1594/PANGAEA.973160

### 2.2. Automatic detection of fin whale vocalizations

**Figure 2.**
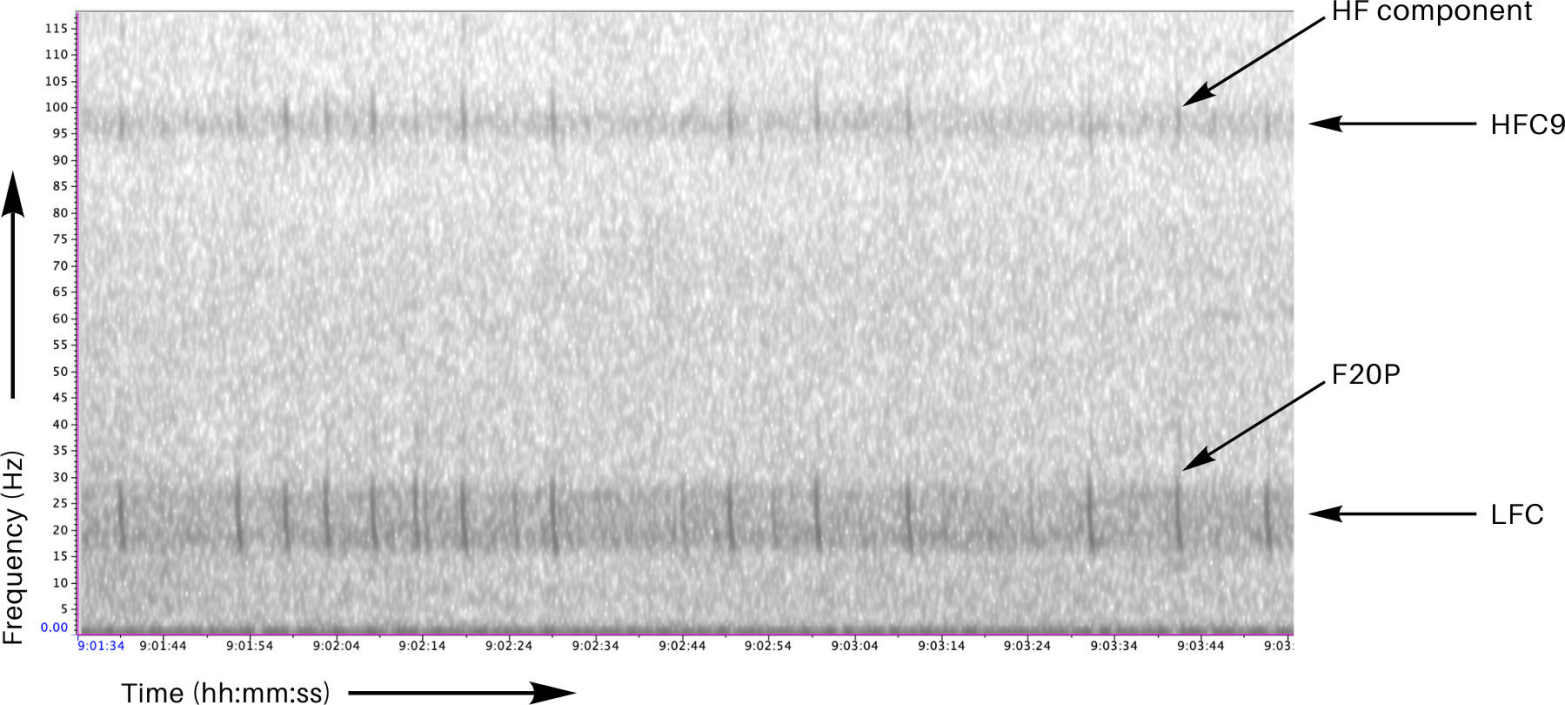
Fin whale call spectrograms of recordings from 28 March 2013 at the Greenwich Meridian (R7). Spectrogram is showing 20-Hz pulses (F20P), the resulting low-frequency chorus (LFC), as well as the simultaneous higher frequency (HF) component centred at approximately 99 Hz, resulting in the 99-Hz chorus (HFC9). Spectrogram parameters: Hanning window with a window size of 5000, discrete Fourier transform size of 8192 and an 80% overlap.

All available passive acoustic datasets were processed using the automated detector developed by Schall & Parcerisas [49]. This detector operates on a threshold-based approach, requiring signal-specific metrics to exceed predefined thresholds to identify fin whale vocal activity. It is designed to identify fin whale 20-Hz pulses, as well as low-frequency and high-frequency choruses generated by spectrally and temporally overlapping 20-Hz pulses and their respective HF components (see figure 2). For chorus detection, the detector calculates three metrics: signal-to-noise ratio (SNR), power spectral density slope (PSD Slope) and power spectral density area (PSD Area). These metrics are compared against predefined thresholds to identify the presence of low- and high-frequency choruses. For 20-Hz pulse detection, the detector evaluates the features kurtosis, temporal and spectral SNR, and signal bandwidth. It employs a decision tree approach that applies multiple thresholds to filter potential detections. The selection of optimal threshold values was performed by maximizing true positive detections while minimizing false positives in two different test datasets. For the chorus test dataset, true positives referred to the correct identification of chorus presence within 5 min audio files. Whereas for the pulse test dataset, true positives referred to the correct identification of time-stamped individual 20-Hz pulses. Manual annotations served as the reference for identifying true positives and false positives.

For low-frequency chorus detection in the frequency band 17−25 Hz and high-frequency chorus detection in the frequency bands 84−87 Hz (86-Hz chorus) and 96−100 Hz (99-Hz chorus), we employed the detection metrics and the corresponding threshold values that lead to the optimal balance between true (TPR) and false positive rates (FPR), as determined by the test dataset used by Schall & Parcerisas [49]. These detection metrics and thresholds, along with the corresponding values of TPR and FPR (estimated in the test dataset from Schall & Parcerisas [49]), can be found in table 2. The chosen detection method for the low-frequency chorus was the SNR metric. For each audio file, the SNR is calculated by estimating the spectral energy within the 17−25 Hz band and comparing it to the noise level in adjacent frequency bands. The median noise level is used to exclude high-energy transient sounds. For the high-frequency choruses, the PSD Area metric was chosen as the optimal detection method. These methods were found to be most effective for detecting the respective choruses in the analyses by Schall & Parcerisas [49]. To minimize the detection of environmental noise within the high-frequency chorus frequency bands, detections of the high-frequency choruses were only counted when the low-frequency chorus was detected concurrently, since the HF component is thought to only occur in relation with the 20-Hz pulses in the SO [12,26].

**Table 2.** Threshold values for the respective signal detection methods and corresponding true positive rates when allowing for the stated false positive rates as estimated by Schall & Parcerisas [49]. Note that the detection metrics for the choruses are independent algorithms that yield three independent indications of chorus presence, while the detection metrics for the 20-Hz pulses combine to a single detection algorithm yielding a single indication of 20-Hz pulse presence. A multi-step decision was conducted, allowing for fainter 20-Hz pulse detection within a chorus (i.e. lower temporal SNR.2 than temporal SNR), if a certain bandwidth was exceeded and the 20-Hz pulse represents a clear pulse (i.e. a higher kurtosis than the signal’s kurtosis 1.2). SNR, signal-to-noise ratio; PSD, power spectral density; TPR, true positive rates; FPR, false positive rates.

signal type	detection method	threshold value	TPR	FPR
low-frequency chorus	SNR	4	0.89	0.03
86-Hz chorus	PSD Area	0.3	0.93	0.03
99-Hz chorus	PSD Area	0.35	0.76	0.03
20-Hz pulse	signal kurtosis	3.25	0.8	≤0.01
20-Hz pulse	kurtosis product	40		
20-Hz pulse	spectral SNR	9		
20-Hz pulse	temporal SNR	−2		
20-Hz pulse	temporal SNR.2	−7		
20-Hz pulse	signal bandwidth	75		
20-Hz pulse	signal kurtosis 1.2	4		

For the detection of 20-Hz pulses, we also selected thresholds that yielded the optimal balance between TPR and FPR (table 2), as outlined in Schall & Parcerisas [49]. This selection was applied across various detection metrics, including the signal’s kurtosis, kurtosis product, temporal SNR, spectral SNR and bandwidth.

For consistency and to ensure comparability, the thresholds for both chorus and pulse detection were applied uniformly across all recording positions analysed in this study.

#### 2.2.1. Manual post-processing of detector results

To assess the presence of low- and high-frequency choruses and pulse detections at each recording position, we conducted a series of checks on the data for each recording location.

Specifically, we randomly selected 10 days for each recording position to manually assess the presence of both low- and high-frequency choruses. From those 10 days, four days were further used to check the 20-Hz pulse detections. Moreover, we revised outlier days, which appeared as temporal exceptions in the timelines exceeding the 1% FPR threshold for 20-Hz pulse detection or the 3% FPR threshold for chorus detection, respectively. This revision of outliers encompassed both detection types across all recording positions and only addressed cases that were not initially covered in the random checks.

Due to an unexpected high number of pulse detections at recording position R5, we extended the evaluation efforts specifically for this recording position, by examining 7 random days per month. All recordings were evaluated in RavenPro 1.6 (The Cornell Lab of Ornithology, Center for Conservation Bioacoustics in Ithaca, NY) using smoothed spectrograms in a Hanning window with a window size of 5000, discrete Fourier transform size of 8192 and 80% overlap.

### 2.3. Analysis of high-frequency components

Since 20-Hz pulses were detected at R1 and R7 only, the analysed recording files of these two locations were filtered for the highest 20-Hz pulse SNR values in combination with 86-Hz chorus or 99-Hz chorus presence, respectively. The ten 10 min files with the highest SNR values and high-frequency chorus presence per recorder position R1 and R7 were used to analyse the detailed frequency content of the HF components detected in this study. In addition to the R1 data from 2013, data from 2019 at R1 were also processed in the same manner to facilitate a comparison between those years to explore the consistency of the peak frequencies over time. Recording snippets from Juan Fernandez [25], the Western Antarctic Peninsula (WAP) [30] and the South Orkney Islands [10] were provided by the respective study authors upon request and were analysed in the same manner as the data of this study. This allowed for a direct comparison of different HF components also from sites outside the study area (see electronic supplementary material, figure S3, for the respective recording positions). To enable direct and optimal comparison, all audio files were decimated to 250 Hz, and analyses were performed in Raven Pro 1.6 (The Cornell Lab of Ornithology, Center for Conservation Bioacoustics, Ithaca, NY) in a Hanning window, with a fast Fourier transform of 256 and 80% overlap. For all encountered HF components, the peak frequency, representing the frequency at which the peak amplitude occurs within the selection box (so-called robust measurement [50]), was measured by drawing selection boxes around the rough HF component’s frequency limits in order to minimize the analysts’ bias. Additionally, a Kruskal–Wallis test was conducted in RStudio (v. 2023.06.1+524 [51]) to assess differences in HF components across sites, followed by a Dunn’s *post hoc* test for pairwise comparisons.

### 2.4. Sound propagation modelling

To estimate the range over which vocalizing fin whales were detected in this study, sound propagation modelling was employed. The site-specific sound propagation of 20-Hz pulses at recording positions R1 and R7 was inversely modelled in three dimensions using the software dBSea (dBSea Ltd, v.2.2.5, developed by Marshall Day Acoustics and Irwin Carr Consulting, UK). For this purpose, the vocalizing virtual whale was assumed to be situated at the respective recorder positions and depths (see table 3 for details), while the received levels (RLs) were calculated for 10.7 m, the depth of the model grid points best representing the assumed fin whale calling depth of 15 m [53]. The virtual sender was assumed to signal omnidirectional, with a source level (SL) of 180 dB re 1 µPa (based on a conservative approach using SLs of previous reported fin whale 20-Hz pulses; [54–57]).

**Table 3.** Information on the recorders, the virtual senders and settings used for sound propagation modelling using the dBSea software (dBSea Ltd, v.2.2.5, developed by Marshall Day Acoustics and Irwin Carr Consulting, UK) for recording positions R1 and R7.

	R1 (Elephant Island)	R7 (Greenwich Meridian)
gains and sensitivity levels of recorder	set gain 48 dB, sensitivity level of 192.6 dB at 251 Hz	set gain 24 dB, sensitivity level of 192.6 dB at 251 Hz
location	61 0.88° S, 55 58.53° W	59 2.82° S, 000 5.78° E
water depth at location	320 m	4600 m
receiver depth	215 m	1020 m
water temperature (summer)	0.8°C	−0.3°C
sender depth	15 m	15 m
sediment type	sand [52]	mud [52]
number of grid points in *x*-directions (resulting step size)	2000 (354.4 m)	610 (353.1 m)
number of grid points in *y*-directions (resulting step size)	2000 (343.4 m)	635 (346.8 m)
number of grid points in *z*-directions (resulting step size)	500 (10.7 m)	525 (10.7 m)
source solution radial slices (resulting slice step angle of source)	100 (3.6°)	100 (3.6°)
source solution range points (resulting range steps of source)	500 (354.2 m)	620 (354.7 m)

While Burkhardt *et al*. [11] used silt as a sediment type based on Diekmann & Kuhn [58] for their sound propagation modelling at Elephant Island, the same location as R1 in our study, the sediment types for R1 and R7 were chosen according to the latest information from Jerosch *et al*. [52]. The sediment types were implemented in the model by using the software’s default settings after Jensen *et al*. [59] for the respective sediment properties (see table 3 for details on the recorder and chosen software settings). Water temperature was determined calculating the water column mean temperature of the location closest to the recording positions, using the austral summer statistical means from 2005 to 2017 with a 1° resolution from the World Ocean Atlas [60].

The models for R1 and R7 were solved for normal modes and, due to different spatial extents of the bathymetry data, respective grid sizes were chosen to ensure comparable resulting step sizes into all three dimensions (i.e. resolution in *x*, *y* and *z* direction; see table 3 for the calculated grid points and the respective resulting step sizes). Further, a source solution of 100 radial slices was chosen for both models (R1 and R7), while the range points were adjusted according to the respective grid sizes. For the slice step angles, range points and resulting steps see table 3.

For comparison of the modelled RL at the recording positions, the received sound pressure levels (SPL_rms_ (dB re: 1 µPa)) of the detected 20-Hz pulses in this study were determined in MATLAB (v. 2022b [37]), by extracting single audio snippets based on the 20-Hz pulse detections and filtering for frequencies between 15 and 26 Hz.

## 3. Results

A total of 65 640 h of recordings collected from 10 positions over a period starting December 2012 until November 2013, spanning 2735 days, were analysed to assess the acoustic presence of fin whales. Out of these recorded days, 150 randomly selected days (15 per recording position) were post-processed by human analysts to verify automated detections, with 7 additional random days per month specifically examined for R5 due to the unexpectedly high number of pulse detections at that location. Subsequent manual examinations on these days revealed that the detections were caused by noise, as no fin whale vocalizations were found in the R5 recordings. Consequently, this recording location is not considered hereafter.

### 3.1. Chorus detection

**Figure 3.**
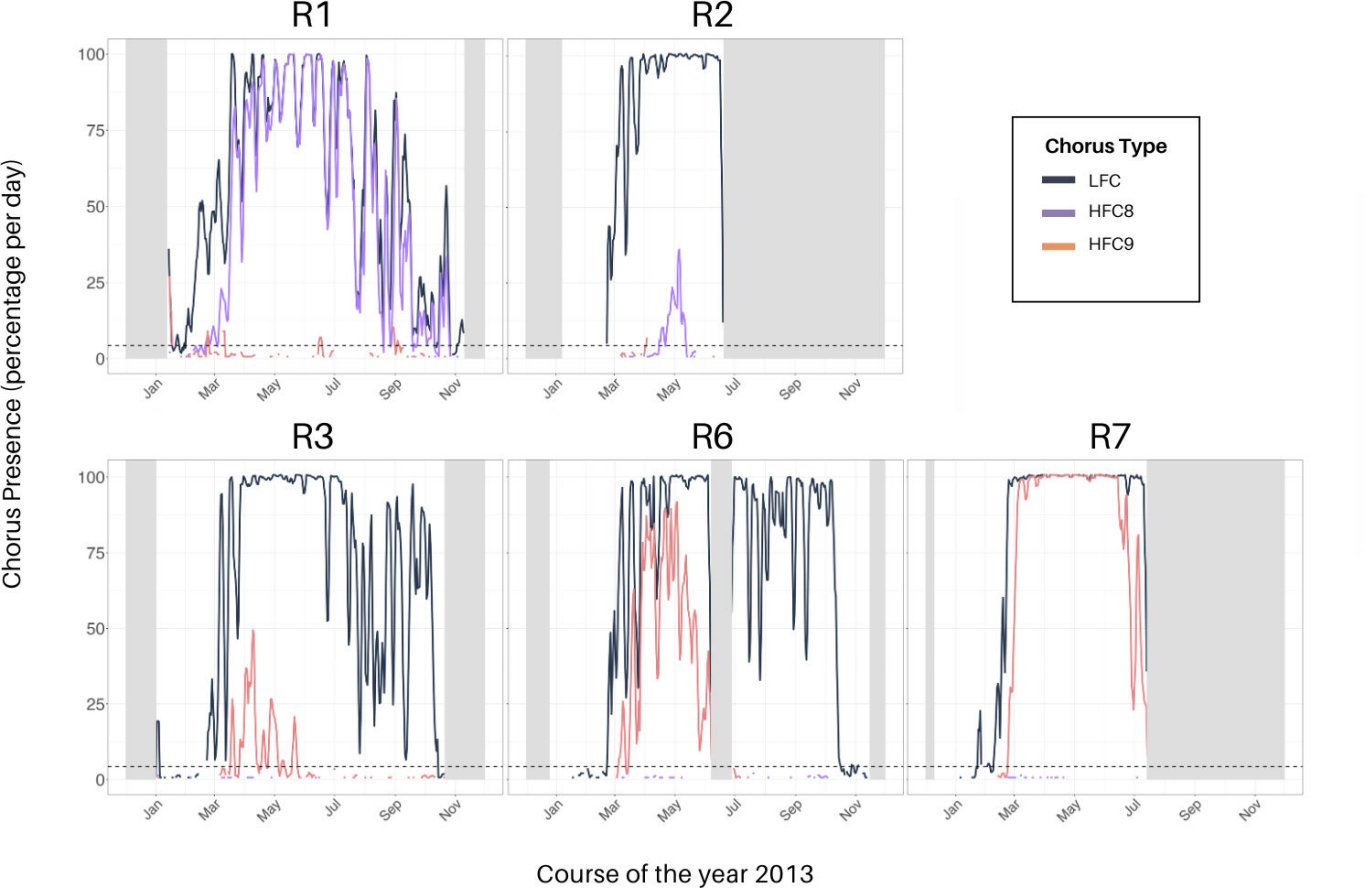
Line plots representing the percentage of files containing low- and high-frequency chorus per day over the course of the year 2013. Recordings consisted of 144 10 min files per day. Low-frequency chorus (LFC) is displayed in dark grey, 86-Hz chorus (HFC8) in purple and 99-Hz chorus (HFC9) in orange. The lines were computed with a three-day running mean to smooth out spikes for a better overview. Dashed horizontal lines indicate the false positive rate of 3%, representing 4.3 recording files per day, as calculated by Schall & Parcerisas [49]. The grey bars indicate time periods where no data were available. The low- and high-frequency chorus presence patterns of R3, R6 and R7 are representative of patterns observed also at R4, and R8–R10; thus, the respective plots will not be discussed further but are available in electronic supplementary material, figure S1.

The low-frequency chorus was detected at all recording positions. Daily chorus presence showed an overall seasonal pattern of low-frequency chorus presence from end of February throughout mid-October, corresponding to periods when recordings were available (see figure 3). Low-frequency chorus was present at R1 throughout November, with a drop in September. At R7, low-frequency chorus was often measured continuously in the period from the end of February to the end of June, while at monitoring sites R1–R4, R6 and R8–R10 this was the case from mid-March to the end of June (see figure 3 and electronic supplementary material, figure S2).

In contrast to the low-frequency chorus presence, the overall presence pattern of the high-frequency chorus was shorter, revealing a delayed onset around the beginning of March. At R2, a less prominent high-frequency chorus presence started with an even greater delay between low- and high-frequency chorus onset at the end of April. At R1 and R7, following the delayed onset, the high-frequency chorus was recorded continuously throughout most days, often revealing identical patterns to the low-frequency chorus (see figure 3).

**Figure 4.**
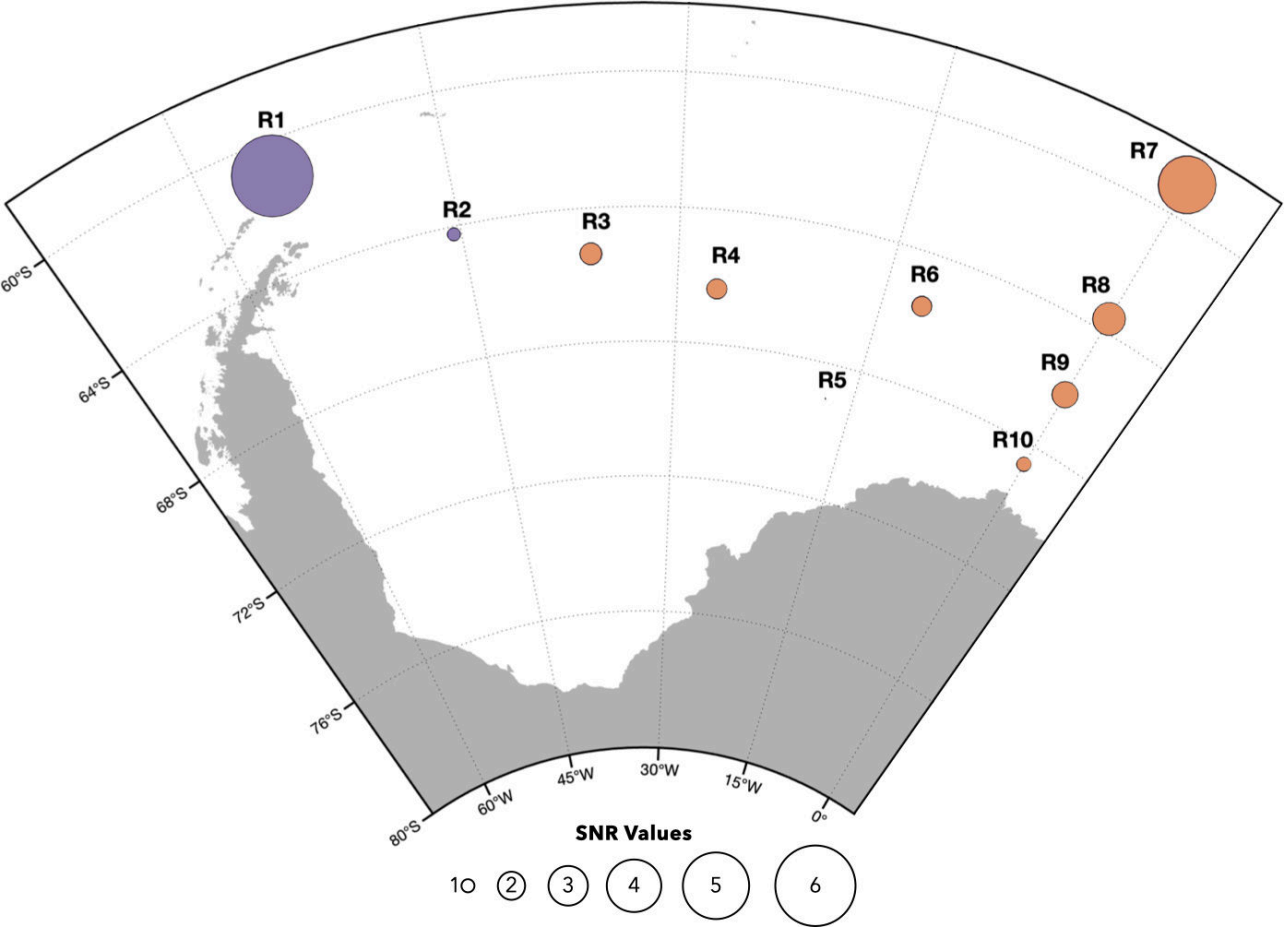
Map indicating the relative differences of the signal-to-noise ratios (SNRs) in dB of the two detected high-frequency choruses at the geographical locations of the ten acoustic recorders used in this study (86-Hz chorus is displayed in purple and 99-Hz chorus in orange). The displayed SNRs were computed as the average of SNR values determined by the automated chorus detector over the respective recording periods. Values were multiplied by 12 to create suitable sized markers (1–6). SNR values of chorus detections below the false positive rate of 3% were neglected. Map was generated with M-MAP in MATLAB [37].

While both previously reported high-frequency choruses at 86 and 99 Hz were present in our data, there appears to be a geographical boundary in their occurrence within the ASSO (see figures 3 and 4). Strikingly, the 86-Hz chorus was only detected in considerable amounts at R1 and R2, while 99-Hz chorus was only detected in considerable amounts at locations R3, R6 and R7. In individual files, 86-Hz chorus was also detected at R3, R6 and R7, as well as 99-Hz chorus at R1 and R2. However, these daily percentages either fall below the FPR of 3% or presence could not be confirmed during manual cross-checking the files and therefore not considered any further.

In addition to the already described differences in chorus presence, we also observed differences in the annual average SNR values. Highest annual SNR values were measured at the northernmost recording locations R1 and R7 with values of 6.08 and 4.31 dB, respectively, and continuously decreasing values towards the southern locations. This can be seen best in the recording transect along the Greenwich Meridian (R7–R10), where the lowest calculated average was 1.06 dB at R10. Additionally, a decrease in SNR values is not only observed along the longitudinal gradient, but with a latitudinal influence from locations R1 and R7 toward the central region of the Weddell Sea.

### 3.2. Detection of 20-Hz pulses

**Figure 5.**
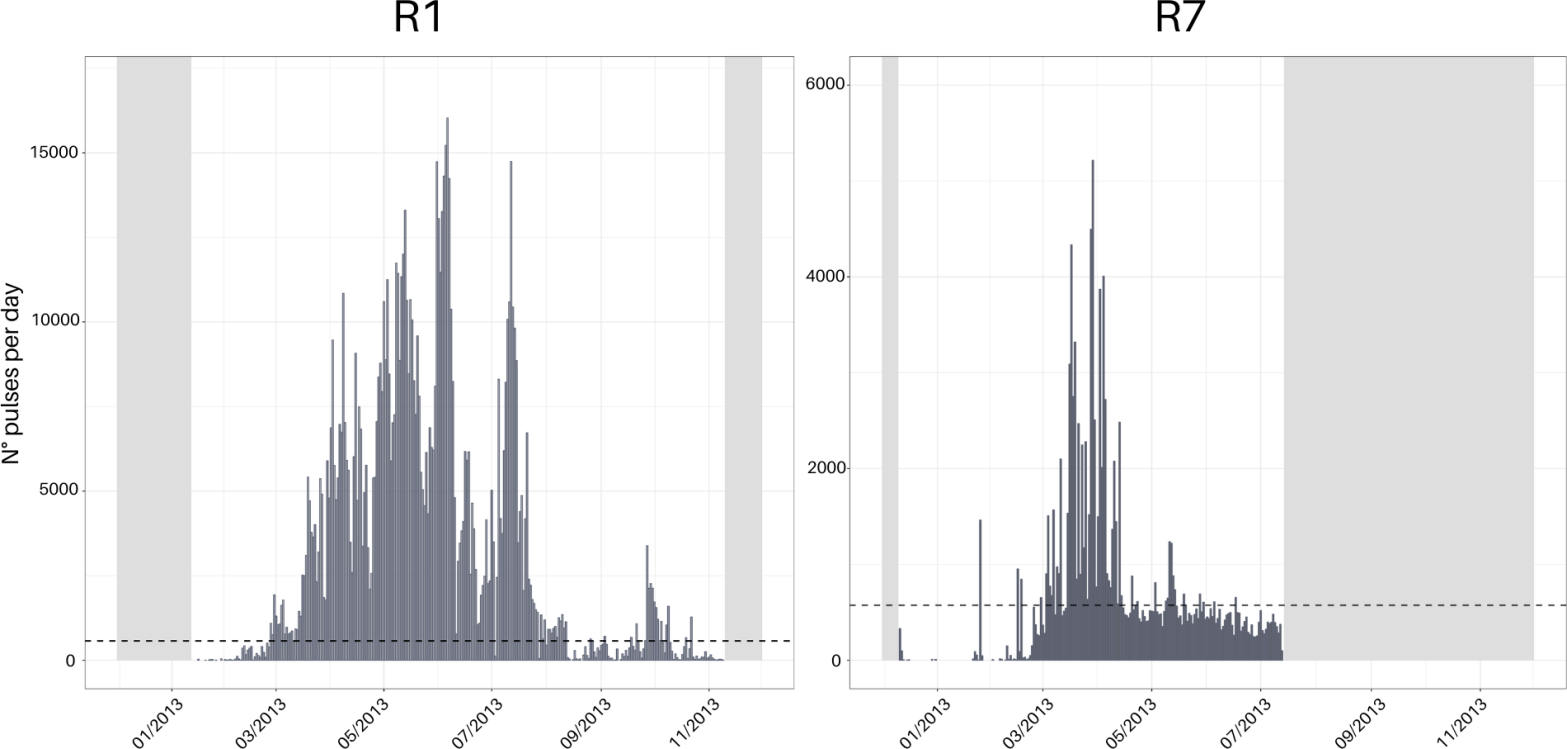
Barplots representing the number of detected fin whale 20-Hz pulses per day over the recording periods for recording positions R1 and R7. Dashed lines indicate the false positive rate of 1%, representing 575 pulses per day, as estimated by Schall & Parcerisas [49]. Note the difference in the scaling of the *y*-axis. No 20-Hz pulses were detected at the remaining recording positions. (Light) grey bars indicate periods where no data were available.

Fin whale 20-Hz pulses were detected and confirmed at R1 and R7 only, revealing a disparity in local call activity between the recording positions (see figure 5). At R1, 20-Hz pulses were detected from the beginning of March throughout the beginning of August, with an additional smaller peak at the end of September and beginning of October. The temporal maximum occurs at the beginning of June with 16 028 detected calls on 6 June 2013, before the call activity decreases and peaks again in July. Compared to R1, the call activity at R7 was detected during a shorter period of the year, and 20-Hz pulse detections were overall less abundant. Calls were occasionally detected on days in February, May and June, whereas the majority of calls were detected in March and the first half of April with a peak on the 29 March 2013 with a total of 5215 20-Hz calls detected.

### 3.3. High-frequency components

**Figure 6.**
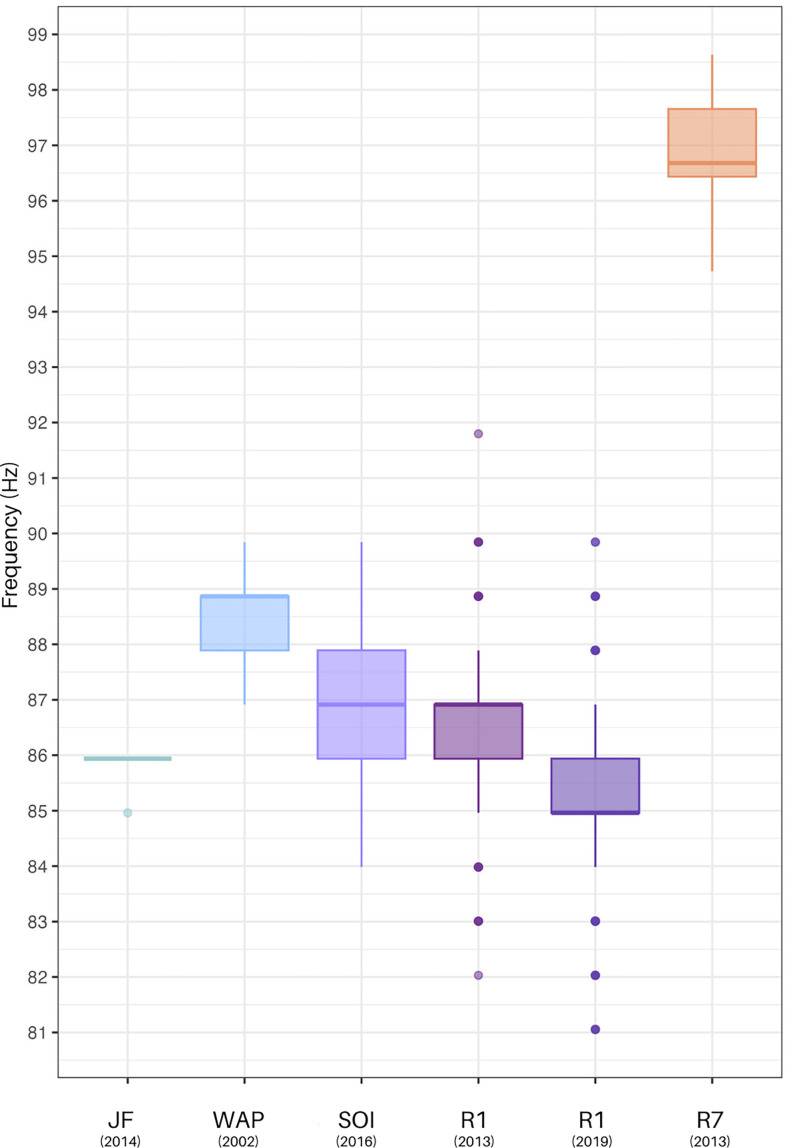
Boxplots comparing the peak frequencies of high-frequency components from this study (R1 (2013) *n* = 225, R1 (2019) *n* = 220, and R7 *n* = 212), as well as recording snippets from Juan Fernandez (JF), Chile recorded in 2014 (*n* = 7 [25]), Western Antarctic Peninsula (WAP) in 2002 (*n* = 21 [30]) and South Orkney Islands (SOI) in 2016 (*n* = 126 [10]).

Peak frequencies of the detected HF component revealed clear differences between the 99- and the 86-Hz chorus but also indicate a broader range of peak frequencies for the HF component considered as 86-Hz chorus across the different locations in the ASSO, South Pacific and South Atlantic (figure 6). While the means of the western locations (Juan Fernandez, WAP, South Orkney Islands and R1) were situated between approximately 85 and 90 Hz, the HF component at R7 was characterized by a clearly higher peak frequency with a mean at 96.82 (±0.86) Hz.

Among the western locations, the means of peak frequencies of Juan Fernandez at 85.79 (±0.37) Hz, the South Orkney Islands at 86.58 (±1.26) Hz and R1 (2013) at 86.48 (±1.25) Hz, as well as R1 (2019) at 85.26 (±1.5) Hz showed high similarities, while off the WAP a higher mean value at 88.45 (±0.85) Hz was measured.

The boxplots in figure 6 showing the detailed distribution of measured frequencies reveal highest overall variabilities with maximum values of approximately 92 Hz and minimum values of approximately 81 Hz at R1 (2013 and 2019)—a range encompassing all analysed peak frequencies from Juan Fernandez, WAP and the South Orkney Islands. However, peak frequencies at R1 (2013 and 2019) result in smaller interquartile ranges than off the South Orkney Islands, whereas the medians of R1 2013 and the South Orkney Islands show more overlap than the medians from R1 2013 in comparison to 2019, representing the lowest measured median (see electronic supplementary material, table S2, for details). Moreover, the Kruskal–Wallis test revealed significant differences across sites (*χ*^2^ = 571.61, *p* < 2.2 × 10^−16^). The pairwise comparisons provide more detailed insights into the differences between sites. As shown in the boxplots, R7 stands out as the most distinct site, displaying significant differences with all other locations. R1 from 2019 and the WAP also show differences from other western sites, although less pronounced (see electronic supplementary material, table S3 for detailed results).

### 3.4. Sound propagation modelling

**Figure 7.**
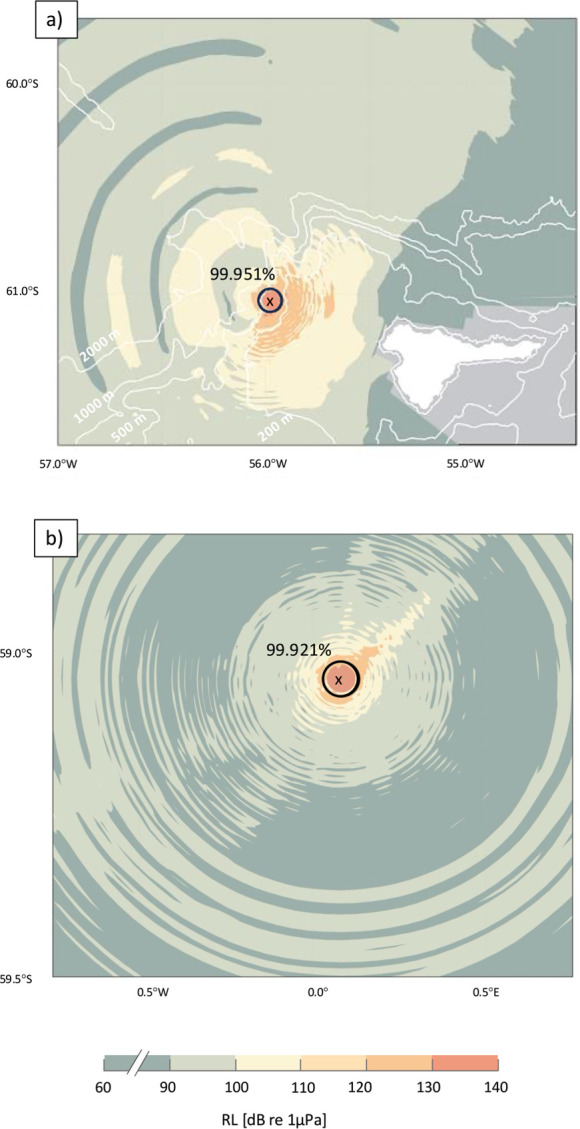
Modelled received sound pressure levels calculated for locations (*a*) R1 off Elephant Island and (*b*) R7 on the Greenwich Meridian. Received levels represent the sound pressure levels at a depth of 10.7 m as received from a virtual source placed at the recorder’s position at (*a*) 215 m depth and (*b*) at 1020 m depth in accordance with the respective recorder depths and assuming a source of SL = 180 dB between 18 and 22 Hz. This set-up serves as a model proxy for the real, reverse situation, i.e. a source situated at 10.7 m and the recorder deployed at 215 or 1020 m depth, respectively. The black circle indicates a 3 km radius around the recording positions R1 and R7. Map created in MATLAB [37,61,62].

While at R1 the sound propagation modelling with dBSea shows a highly anisotropic detection range, due to local bathymetric features, it reveals a relatively isotropic RL distribution at R7 when assuming a source at 10 m depth with a SL at 180 dB re 1µPa (virtual fin whale; see figure 7 for colour coded modelled RL distribution at the respective recording positions).

Based on the model output and the calculated SPL_rms_, the average RL was calculated for a maximum distance of 3 km (indicated by the black circles in figure 7, resulting in RL values of 115.75 and 115.86 dB re 1 µPa for R1 and R7, respectively). Thus, the SPL_rms_ calculation of the detected 20-Hz pulses in this study revealed that presumably 912 674 calls (with RL > 115.75 dB re 1 µPa), making up 99.951% of all detected calls at R1, originate from within a 3 km radius around the recording position. In comparison, 127 016 calls (with RL > 115.86 dB re 1 µPa) of all detected pulses, accordingly 99.9%, presumably originate from within a 3 km radius at R7.

## 4. Discussion

The detection methods used to automatically detect fin whale acoustic presence relied on various metrics including but not exclusively simple SNR measurements. While commonly and effectively used [30,31,63], the calculation of SNR has limitations in environments where background noise overlaps with the target frequency range, potentially leading to false positives or missed detections. However, it is important to highlight that this detector was specifically designed to minimize false positives and avoid overestimating fin whale presence. As Schall & Parcerisas [49] note, the goal was to reduce false positives and prevent the misclassification of similar sounds, ensuring that fin whale presence is not overrepresented. This approach may result in an underestimation of fin whale presence in noisy environments, but it effectively prevents the overestimation of acoustic presence. Therefore, we are confident that detected fin whale acoustic presences are not caused by noise to a greater extent (>1% accepted FPR), allowing us to reliably interpret these results in an ecological context in the following paragraphs.

### 4.1. High-frequency components as population identifier

We found a clear geographical distribution of HF components in the Weddell Sea, with main presence of 99-Hz chorus along the Greenwich Meridian and 86-Hz chorus detected at the westernmost locations off the WAP. However, our study spans only 1 year of data (2013; with additional analysis at R1 from 2019), which may limit our ability to detect annual variability in the geographic distribution of these HF components.

Acoustic variation across different geographical regions does not necessarily indicate genetic divergence. SHFWs are thought to represent a single circumpolar genetic population and show low genetic differentiation across regions [64,65]. At the same time, as this study shows, SHFWs differ regionally in their acoustic characteristics.

Acoustic divergence in calling behaviour in the absence of significant genetic differences between calling populations has been found in species such as North Pacific sperm whales [66] and bird species like *Campylopterus curvipennis* and *Sylvia atricapilla* [67,68]. Hatch & Clark [17] also observed acoustic differences in NHFW calls and suggested that these may represent intraspecific variation that is too recent to be detectable in the genome [17]. This advocates that acoustic traits can be shaped by the social environment and learning rather than by genetic differences [66]. Cultural transmission, the transfer of shared behaviour or information through social learning, however, has the potential to affect the gene distribution. Culture includes vocalizations as well as habitat use, migration patterns, foraging strategies, prey selection and social behaviour [69,70]. Transmission of culture can occur directly from parents to offspring (vertical) and between unrelated individuals from the previous to later generation (obliquely [71]). One very prominent example is killer whales (*Orcinus orca*), where stable cultural traditions within ecotypes, including distinct vocal repertoires and prey choice, have led to functional gene evolution up to a level of ecological speciation [72–75]. Moreover, in various bird and cetacean species with less advanced ongoing speciation, horizontal cultural transmission (between unrelated individuals or even between neighbouring populations within the same generation) is found [76–79]. Southern Hemisphere humpback whales are managed as distinct breeding stocks characterized by their stock-specific song [14,80]. Despite gene flow and horizontal transmission of specific songs still occurring on shared summer feeding grounds [13,81], genetic differences between these breeding stocks are already detectable [82]. While cultural transmission may eventually be reflected in the genome, it seems cultural divergence, not genetic variation, drives acoustic differences first.

Considering this, bottleneck events may impact not only the genetic diversity of a population but also its cultural traits. In bird species, population size has been shown to correlate with acoustic diversity, which tends to decline following decimation events that isolate subpopulations or reduce population numbers [83–86]. Severe decimation events may also lead to cultural conformism and the loss of traditional knowledge, including the loss of traditional foraging grounds [87]. This has been suggested as a factor limiting the recovery of species like the North Atlantic right whale [88]. Thus, historical and industrial whaling may have affected subpopulations of whales, without necessarily reducing their genetic diversity. For instance, studies on North Atlantic fin whales and Southern Hemisphere humpback whales found no significant impact of whaling on genetic diversity [89,90]. And although the extensive depletion of SHFW populations might not be detectable through genetic data either, it may have severely disrupted social structures and cultural groups, potentially leading to cultural losses that cannot be identified through genetic analysis alone. Therefore, from a conservation perspective, it may not only be important but possibly more effective to focus on identifying and protecting culturally distinct subpopulations before prioritizing genomic differentiation [66].

While SHFW are currently considered a single circumpolar genetic population, they seem to represent several distinct acoustic subpopulations, with divergence too recent or subtle to be detectable in the genome. Given that differences in acoustic characteristics provide a reliable identifier for acoustic subpopulations, differences in acoustic behaviour like song characteristics and HF components can offer a suitable alternative for defining acoustic subpopulations [91]. However, in order to employ differences in acoustic characteristics as a robust method to identify acoustic populations, the acoustic signal needs to contain at least one cue that is stereotyped by this cultural group, ideally remaining stable over multiple years. Baleen whale song characteristics such as INIs in fin whale song are commonly used to differentiate between acoustic populations (e.g. [20,92,93]). The duration of INIs can seasonally change from shorter to longer patterns towards the beginning of the migration to breeding grounds [25,94] and hence might not provide a sufficiently stable characteristic to determine population identity. Furthermore, identification of clear INI patterns can be particularly challenging in regions with high fin whale densities, given the overlap in calling bouts [11]. Unlike INIs, HF components may offer a more reliable and temporally stable cue for population identification, if they remain consistent over multiple years.

Previous work reported that the frequency of the fin whale calls is decreasing over years, which seem to render the HF component less suitable as population identifier. A steady decrease in frequencies over several years was reported in HF components produced by North Atlantic fin whales [20], in 20-Hz pulse song and the 99-Hz HF component of SHFW [35,95], as well as in song of other baleen whales, such as blue and pygmy blue whales [35,96,97]. When comparing reanalysed HF components recorded off the WAP between 2002 and 2019, our results show a similar decline (figure 6). Širović *et al*. [30,31] detected a HF component at 89 Hz (88.45 ± 0.85 Hz) further west off the WAP in 2002 recordings. Burkhardt *et al*. [11] detected a HF component around 86 Hz (85.6 ± 1.5 Hz) off Elephant Island (R1 in this study) in 2013, while Buchan *et al*. [25] recorded a similar HF component at 85.79 ± 0.37 Hz off Chile in 2014. This was suggested to reflect a downward shift in frequency, similar to that observed in other baleen whale populations [11,25,33]. However, our data from R1 in 2013 and 2019 reveal an overall range of HF components that include the 89-Hz HF component from Širović *et al*. [30,31]. Additionally, the reanalysed data from the South Orkney Islands in 2016 [10] show a similar median to the R1 data from 2013, while the interquartile range extends to higher peak frequencies, more similar to values observed off the WAP in 2002. This may indicate a less steady frequency decline in these regions over time or suggest the presence of an acoustic population with a broader HF component range in these waters. Our reanalysis at Juan Fernandez and the WAP was based on a relatively small sample size and requires more in-depth evaluation. Further analysis of PAM data from areas such as the South American east coast and both African coastlines would be valuable before drawing final conclusions.

### 4.2. Main habitats and migration of SHFW across the ASSO

This study reveals seasonal SHFW occurrence in the ASSO starting in late February (late austral summer) and continuing through austral autumn until October (austral winter), consistent with previous findings [11,29,30,98,99]. Notably the low-frequency chorus was recorded with a high daily percentage at all locations (except R5), while the high-frequency chorus showed a weaker overall pattern with a higher daily presence at R1 and R7. This pattern is corroborated by the calculated relative SNR (figure 4), which shows higher values towards the northeastern and northwestern edges of the ASSO. Moreover, 20-Hz pulse detection was limited to R1 and R7, and the sound propagation modelling additionally further indicates that >99.9% of all detected 20-Hz pulses were produced within a radius of 3 km around the respective locations. The results align with the current understanding of SHFW being a pelagic species that is negatively correlated with sea-ice and the general distribution north of 60° S [30,100–102]. Our findings of 20-Hz pulses at the northernmost locations only confirm reported high habitat suitability for SHFW along and between the southern boundary of the Antarctic Circumpolar Current and the southern Antarctic Circumpolar Current Front [102–106]. Furthermore, exceptionally high densities of Antarctic krill (*Euphausia superba*) can be found in regions east and west of the Weddell Sea, respectively, explaining the acoustic presence predominantly towards our recording positions R1 and R7. These regions are characterized by oceanographic features enhancing productivity thereby increasing prey availability for SHFW [107–109]. In particular, Elephant Island (R1), the location with the highest number of 20-Hz pulse detections, is a confirmed and important feeding area not only for SHFW but also for other baleen whale species [11,103,110,111]. The variability in fin whale 20-Hz pulse detections in this area (figure 5) may reflect fluctuations in krill patch quality that might change over the season. Different whale species may prefer different krill demographics, and fin whales have been associated with large (>45 mm), mature Antarctic krill located offshore [111]. Additionally, foraging efficiency in rorquals, such as blue whales, has been shown to depend on prey density [112,113], which may also vary seasonally. These changes in prey demographics and density could drive movement patterns, with whales moving in and out of the region. Such movements, including groups feeding and departing, were observed and could contribute to the observed variability in 20-Hz pulse detections (E.B. 2024, personal communication).

#### 4.2.1. Detection ranges of calls

The high-frequency chorus showed similar but weaker daily and delayed seasonal patterns compared with the low-frequency chorus. One possible explanation could be that the HF component is intended for communication over shorter distances than the fin whale 20-Hz pulses and is therefore not always detectable when low-frequency chorus is present. Simon *et al*. [28] found that for 20-Hz pulses and HF pairs, the HF component has a lower amplitude. This would imply that at the beginning of February, when only the low-frequency chorus is present in our data, fin whales are still further away from the recorder. However, visual sightings showed SHFW present as early as January until February in waters off Elephant Island [106], when 20-Hz pulses and the respective chorus are not present in our data. Irregular migration and/or year-round presence in high-latitude feeding areas has been reported for various baleen whale species (e.g. [114,115]). Given that no fin whale song, which is thought to be produced by males only [22], was recorded before February when visual sightings already confirmed fin whale presence, it may be females or juveniles that overwinter in these waters, as has been suggested for other baleen whale species as well (e.g. [116,117]).

### 4.3. Circumantarctic patterns in fin whale high-frequency components

Generally, our overall comparison of HF components spanning a scale from Chile to New Zealand over 17 years reveals five geographical groups, potentially stocks, throughout the SO.

**Figure 8.**
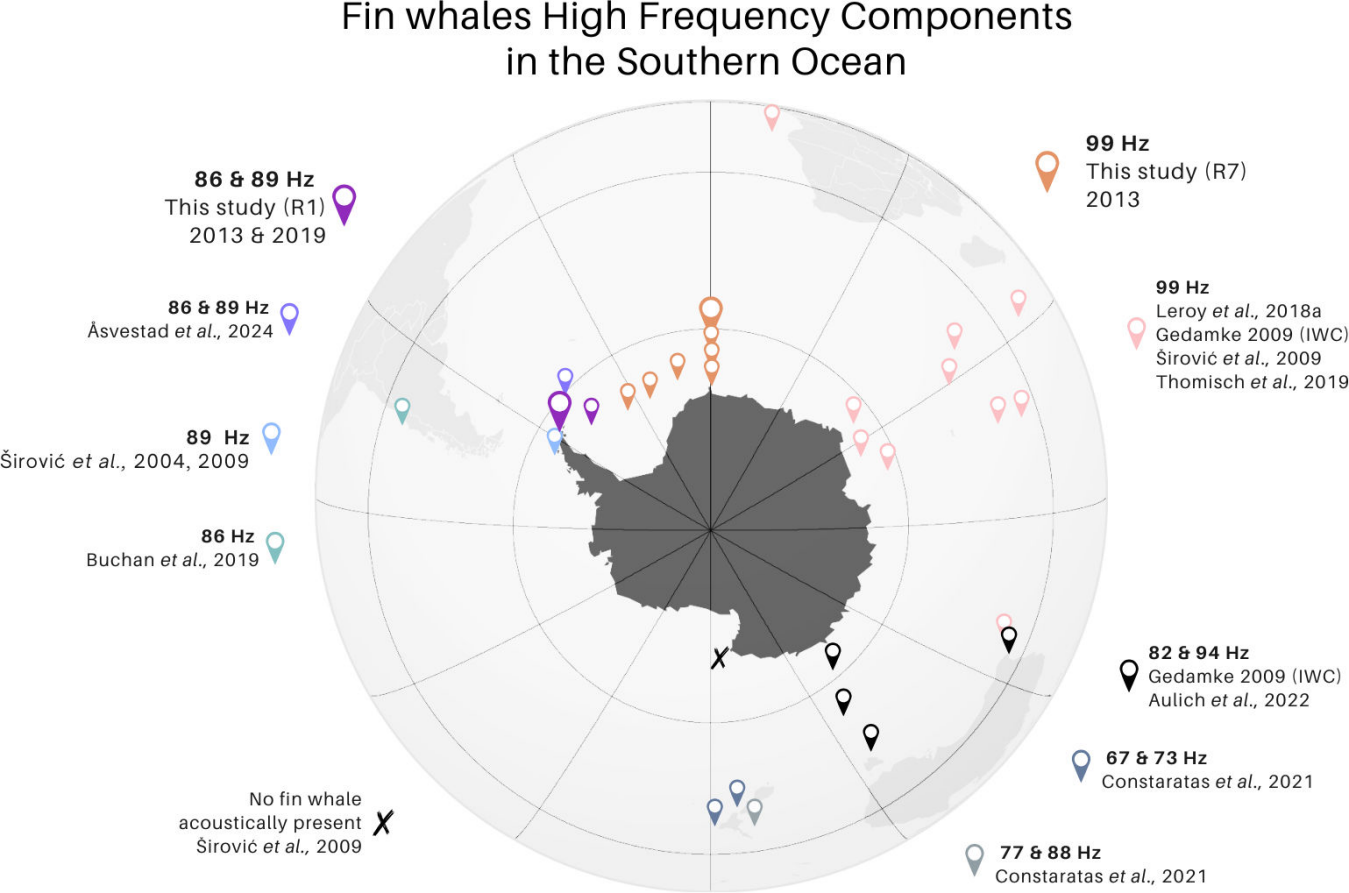
Map illustrating an overview on the different high-frequency (HF) components that were recorded in our and in previous studies in the Southern Ocean [10,25–27,30–32,35]. This map was created on Canva.com as a visual aid in understanding the spatial distribution of fin whale HF components; thus, the accuracy of coordinates of the data points is limited.

Nearly all HF components mentioned in here and shown in figure 8 (and in electronic supplementary material, table S4) were detected with stable but geographically distinct frequencies over several years. Occasional spatial overlaps in HF component occurrence appear, such as off the west coast of Australia. In 2006 data, Aulich *et al*. [34] and Gedamke [27] detected only the 99-Hz component in their recordings. However, the doublet song and corresponding HF component of 82 and 94 Hz were found in recordings from later years at the same recording site. The fact that the two acoustic populations have their latitudinal migratory route from Antarctic feeding grounds along the western or eastern Australian coasts with little longitudinal exchange may explain this slight spatial overlap in HF component types [34]. Interestingly, when examining circumantarctic HF component patterns, doublet songs and corresponding HF components appear only in waters off Australia and New Zealand. Although this song type has not been found elsewhere yet, data from additional locations, comprising both feeding and breeding areas, could help to determine if this phenomenon is truly specific to this region.

The identification of SHFW acoustic populations using the HF components will not only enhance our knowledge on distribution and migratory patterns but enable and facilitate tailored conservation management approaches for the respective populations. Fin whales are not only expected to face further population declines due to climate change by 2100 [118], but they are also increasingly impacted by growing human activities in the SO, such as tourism and fisheries. In the eastern Weddell Sea, the expanding krill fishery poses a rising threat [119], while in the waters off the WAP, fisheries targeting toothfish and increasing tourism are significant stressors [120,121]. This study adds to a growing body of evidence that these areas are vital for endemic as well as seasonal species, and that specific populations rely exclusively on relatively small regions for critical life stages. Our work supports the need for designing and implementing (seasonal) management measures, such as Marine Protected Areas, tailored to conserve and protect the species in this vulnerable region.

## Data Availability

The passive acoustic datasets analysed in this study are available through the PANGEA database (Thomisch *et al*., in preparation). The long-term spectrograms of the analysed recorders can be accessed via the Open Portal to Underwater Soundscapes (OPUS) accessible at CC BY 4.0, AWI 2023 (Thomisch K, Flau M, Heß R, Traumueller A, Boebel O. 2021 OPUS: an open portal to underwater soundscapes to explore and study sound in the global ocean. In *5th Data Science Symposium*; https://www.opus.aq/). Supplementary material is available online [122].
